# *Pochonia chlamydosporia* synergistically supports systemic plant defense response in *Phacelia tanacetifolia* against *Meloidogyne hapla*

**DOI:** 10.3389/fpls.2024.1497575

**Published:** 2025-01-16

**Authors:** Jana Könker, Sanja Zenker, Anja Meierhenrich, Anant Patel, Karl-Josef Dietz

**Affiliations:** ^1^ Plant Biochemistry and Physiology, Bielefeld University, Bielefeld, Germany; ^2^ Fermentation and Formulation of Biologicals and Chemicals, Hochschule Bielefeld - University of Applied Sciences and Arts, Bielefeld, Germany; ^3^ Computational Biology, Bielefeld University, Bielefeld, Germany; ^4^ Center of Biotechnology (CeBiTec), Bielefeld University, Bielefeld, Germany

**Keywords:** plant defense, biotic stress, synergistic effects, systemic plant response, biological control, plant-parasitic nematodes

## Abstract

The network of antagonistic, neutral, and synergistic interactions between (micro)organisms has moved into the focus of current research, since in agriculture, this knowledge can help to develop efficient biocontrol strategies. Applying the nematophagous fungus *Pochonia chlamydosporia* as biocontrol agent to manage the root-knot nematode *Meloidogyne hapla* is a highly promising strategy. To gain new insight into the systemic response of plants to a plant-parasitic nematode and a nematophagous fungus, *Phacelia* was inoculated with *M. hapla* and/or *P. chlamydosporia* and subjected to transcriptome and metabolome analysis of leaves. While the metabolome proved quite stable except for the early time point of 48 h, comparison of the single *P. chlamydosporia* with the combined treatment revealed even larger effects after 6 d compared to 48 h, aligning with the later root infestation by *P. chlamydosporia* compared to *M. hapla*. Simultaneous exposure to both microorganisms showed a stronger overlap with the single *M. hapla* treatment than *P. chlamydosporia*. Changes of transcripts and metabolites were higher in the combined treatment compared to the individual inoculations. The results support the conclusion that *P. chlamydosporia* induces plant defense in a distinct and beneficial manner if combined with *M. hapla* although plant defense is partly suppressed by the endophytic growth. The results tentatively suggested that the application of *P. chlamydosporia* as a biocontrol agent against *M. hapla* can be more effective by supporting these tritrophic interactions with specific additives, such as phytohormones or amino acids in the formulation.

## 1 Introduction

Multiple interactions between plants, microbes, nematodes, and other soil organisms determine the rhizosphere (Hunt et al., 1987; Scheu, 2002; Thies and Grossman, 2023). This network of antagonistic, neutral, and synergistic interactions has moved into the focus of current research, since in agriculture, this knowledge can help to develop efficient biocontrol strategies. Biocontrol reduces the application of environmentally harmful pesticides and at the same time assists in ensuring the agricultural food production that is needed for the increasing world population. A well-known example for such a biocontrol agent is the nematophagous fungus *Pochonia chlamydosporia* (Viaene and Abawi, 2000; Van Damme et al., 2005; Dhawan and Singh, 2010; Askary, 2015; Avelar Monteiro et al., 2017; Manzanilla-López and Lopez-Llorca, 2017). This fungus mainly feeds on nematodes present in the soil but also survives as saprophyte or grows endophytically in a variety of economically important crops (Kerry et al., 1993; Tobin et al., 2008; Hallmann et al., 2009; Manzanilla-López et al., 2011). *P. chlamydosporia* is applied as biocontrol agent against plant-parasitic nematodes such as root-knot nematodes of the genus *Meloidogyne* which are one of the most damaging plant-parasitic nematodes infecting a wide range of crops (Carter, 1985; Jones et al., 2013; Askary and Martinelli, 2015).

*Meloidogyne* penetrates the host root within the first three days after inoculation (Shukla et al., 2018). Secreted effector molecules like proteins and small metabolites initiate and maintain feeding sites and suppress plant defense in host roots (Abad and Williamson, 2010; Mitchum et al., 2013; Shukla et al., 2016). The *Meloidogyne* life cycle consists of six biological stages starting with the eggs and developing through the juvenile stages J1–J4 to the adult stage. J2 juveniles induce giant root galls by modulating plant metabolism and development 5 to 7 days after inoculation (Shukla et al., 2018).

Plants defend themselves against plant-parasitic nematodes by producing small molecules with anti-nematode activity like phenolic compounds, terpenoids, saponins, or glucosinolates, either before or during nematode infection (Abad and Williamson, 2010; Ghahremani et al., 2022). On the other side, auxin conjugates and cytokinin are important components of the plant machinery that is exploited by the nematode during the establishment of the feeding cell. But the detailed molecular mechanisms are still largely unknown (Abad and Williamson, 2010; Shukla et al., 2018). Shukla et al. (2018) identified 1827 significantly differentially expressed genes (DEGs) in infected tomato roots, with predominantly affected functions varying with infection stage. The majority of these genes are involved in cell wall structure (e.g., plant hydrolases, endo-β-1,4-glucanases, pectin acetyl esterase, β-xylosidases, xyloglucan endotransglucosylases, polygalacturonases, and expansins), cell development (cyclin-dependent kinases, mitotic cyclins, tubulin, and actin), primary and secondary metabolism, and defense signaling pathways (plant hormones like jasmonate and salicylic acid).

Endophytically growing *P. chlamydosporia* sensitizes the plants by inducing changes in transcription and metabolism and, thereby, functions as a biological control agent as some isolates induce plant defense through cell wall changes in barley and tomato roots (Escudero and Lopez-Llorca, 2012; Larriba et al., 2015; Schouten, 2016). In barley roots, pathogen-induced genes, e.g., encoding chitinases, methyl esterases, germin A-like proteins and late embryogenesis abundant (LEA) protein, are promoted in the plant response during endophytic growth of *P. chlamydosporia* (Larriba et al., 2015).

*P. chlamydosporia* colonization of barley roots leads to an enrichment of transcripts involved in abiotic stress response, mainly coding for heat shock proteins and proteins involved in phytohormone biosynthesis, in particular auxin, ethylene, salicylic acid, and jasmonic acid. Other changed transcripts relate to plant defense mechanisms like systemic acquired resistance (SAR) (Larriba et al., 2015; Ghahremani et al., 2019; Tolba et al., 2021).

Since both, *P. chlamydosporia* and *Meloidogyne* sp., interact with the plant root, most plant transcriptome analyses focused on infected root tissue and the rhizobiome. In a converse manner, this study aimed to scrutinize the systemic response by exposing the roots of the frequently cultivated cover crop *Phacelia tanacetifolia* (Hydrophyllaceae) to *Meloidogyne hapla*, *P. chlamydosporia*, and a combination of both organisms. Using RNA sequencing and identifying metabolites from *P. tanacetifolia* leaf tissue, the analysis was designed to focus on leaves 48 h and 6 d after inoculation. These time points mark important steps in the life cycles of *P. chlamydosporia* and *M. hapla* in plant root. About 48 h after inoculation *M. hapla* penetrates the plant roots (Shukla et al., 2018) and the blastospores of *P. chlamydosporia* have geminated and initiate their endophytic root growth (Escudero and Lopez-Llorca, 2012; Pentimone et al., 2019). After 6 days, the degree of plant root colonization has increased for *P. chlamydosporia* (Escudero and Lopez-Llorca, 2012; Pentimone et al., 2019) and the *M. hapla*-induced giant root gall has started to form (Shukla et al., 2018).

As described above *P. chlamydosporia* mainly feeds on nematodes and some isolates can induce plant defense by endophytic growth. Due to this tri-trophic interaction this study aimed to address two hypotheses for the effect of combined treatment with *M. hapla* and *P. chlamydosporia*. The first hypothesis is based on the interaction of *P. chlamydosporia* and *M. hapla*. Because *P. chlamydosporia* feeds on *M. hapla* and, therefore, reduces the number of nematodes that can enter the plant roots, transcripts that are known as markers for general abiotic and biotic stresses focusing on ROS-related signaling should be less abundant in *P. tanacetifolia* in the combined treatment compared to the treatment only with *M. hapla* (H1). In contrast, the second hypothesis is based on the interaction between *P. chlamydosporia* and *P. tanacetifolia*. Since some isolates of *P. chlamydosporia* are described to induce plant defense in different ways, indicators for a plant defense response, like pathogenesis-related transcripts and transcripts related plant hormone signaling, could be stronger regulated by synergistic effects in the combined treatment compared to the single inoculations with either *P. chlamydosporia* or *M. hapla* (H2).

## 2 Material and methods

### 2.1 *Meloidogyne hapla* and *Pochonia chlamydosporia* strains

The nematode *M. hapla* inoculum was obtained from galled tomato roots (*Solanum lycopersicum* cv. Moneymaker) grown under greenhouse conditions. The strain is part of the living nematode collection hosted at the Julius Kühn Institute (Braunschweig, Germany), where *M. hapla* juveniles are routinely tested for purity with the species-specific IGS primers JMV1 and JMVhapla according to the protocol of Adam et al. (2007). The washed tomato roots were placed on a 250 µm-sieve (Ø 12.5 cm) on top of a glass funnel (Ø 15 cm) with a silicone hose attached to the base, which is closed with a hose clamp. This setup was placed in a sprayer chamber with a spraying interval of 30 s and 5 min break. Juveniles hatched from the eggs passed the 250 µm-sieve and sunk into the funnel outlet. Every second day the tube clamp was loosened to collect the freshly hatched nematodes in an Erlenmeyer flask to be stored at 6-8°C. The nematode suspension was adjusted to 350 juveniles/ml tap water.

*Pochonia chlamydosporia* blastospores were cultivated according to Uthoff et al. (2023). *P. chlamydosporia* strain Pc001, deposited in the fungal culture collection at the Julius Kühn Institute (Braunschweig, Germany), was originally isolated from surface-sterilized cysts of the sugar beet nematode *Heterodera schachtii* collected from fields located in North Rhine-Westphalia, Germany (Nuaima et al., 2021). To cultivate blastospores, sterile 250 ml flasks with baffled base were filled with 100 ml sterile potato dextrose broth (PDB, 26.5 g/l, Carl Roth GmbH & Co. KG, Karlsruhe, Germany), inoculated with 10^6^ chlamydospores and incubated at 26°C on a rotary shaker at 150 rpm (IKA KS 4000 ic, Staufen, Germany) for five days. PDB liquid culture was filtered through a 5–10-µm Whatman filter (VWR, Darmstadt, Germany) under sterile conditions to separate blastospores from mycelium. The blastospores were washed twice with 0.9% sterile NaCl solution followed by centrifugation (3600 g, 20°C, 10 min) and resuspension in a 0.9% sterile NaCl solution.

### 2.2 Experimental setup under greenhouse conditions and *Phacelia tanacetifolia* harvest

A greenhouse experiment with four treatments was set up to study plant response at transcriptome and metabolome level. To this end, *P. tanacetifolia* plants (cv. Balo, provided by Feldsaaten Freudenberger GmbH & Co. KG, Krefeld, Germany) were grown in sand and vermiculite (4:1, v:v) substrate fertilized with modified Hoagland solution twice a week (full concentration of the fertilizer solution (pH 6.5) contained 5 mM KNO_3_, 2.5 mM Ca(NO_3_)_2_, 1 mM (NH_4_)_2_SO_4_, 2 mM (NH_4_)_2_HPO_4_, 2 mM MgSO_4_, 1 mM KCl, 2 mM NaCl, 2 mM Na_2_SO_4_, 1 mM FeC_6_H_5_O_7_, 0.025 µM H_3_BO_3_, 0.002 µM MnSO_4_, 0.002 µM ZnSO_4_, 0.005 µM CuSO_4_, 0.005 µM MoO_3_). After four weeks, the plants were separated into four groups namely, A) uninfected substrate as control (C), B) *M. hapla*-infested substrate (MH); C) *P. chlamydosporia*-inoculated substrate (PC), and D) in substrate co-inoculated with *M. hapla* and *P. chlamydosporia* (MH+PC). For *M. hapla* infection (treatment B and D), 10 ml tap water with 350 juveniles ml^-1^ were inoculated per pot through five uniformly distributed holes in the substrate, while for *P. chlamydosporia* inoculation (treatment C and D), 10 ml blastospore suspension in 0.9% NaCl (with 3.5x10^7^ ± 3.25x10^6^ blastospores per pot) was provided using similar five holes. Each treatment consisted of twelve replicate pots set up randomly in the greenhouse (temperature 22.4 ± 7.2°C; relative humidity 57.5 ± 15.4%) and watered and fertilized every second day or as required. The plant harvest was carried out 48 h, 6 d and 28 d after inoculation.

To harvest the *Phacelia* plants, the content of a pot was placed in a large bowl and the shoot, and roots were carefully separated from the substrate. The roots were washed quickly in water with as minor damage as possible. Parts of the washed roots and the shoots were separated, packed in aluminum foil and frozen in liquid nitrogen. Two substrate samples were taken to determine (i) the number of colony-forming units (CFU) of *P. chlamydosporia* and (ii) the density of nematodes. The same procedure was repeated for all harvest time points.

### 2.3 *P. chlamydosporia* colony-forming units and plant root colonization

To determine the CFU, the substrate was mixed carefully, and 4 g was removed from each pot, mixed with 10 ml of 0.1% Tween 80, and shaken at 50 rpm for 30 min. The samples were stored for 10 min to allow the substrate to sediment. 10 µl supernatant was taken from each sample and plated on selective PDA medium (Strasser et al., 1996). For each sample three technical replicates were incubated at 23°C for 5 d and fungal CFU per plate were counted.

A representative sample was prepared from each washed root system. For this purpose, 1 cm long fragments were cut from different areas of the roots. These root segments were heated for 20 to 60 min (depending on the thickness of the roots) at 90°C in 10% KOH. After heating, the root segments were rinsed with deionized water and stained in ink-vinegar solution (100 ml blue ink, 100 ml 10% acetic acid solution and 800 ml water) at 90°C for 15 to 20 min (Giovannetti and Mosse, 1980). The root pieces were carefully rinsed again in deionized water until no more ink could be washed out. To quantify fungal structures in the roots, stained root fragments were placed on a microscope slide and covered with lactoglycerol. The quantification was carried out microscopically at 200-fold magnification (McGonigle et al., 1990). The slide was passed over vertically (i.e., perpendicular to the roots) with a crosshair ocular. Each time the crosshairs intersected a root, an intersect was counted. The crosshairs were always aligned vertical to the root. At least 100 intersects were counted and the degree of colonization was expressed as the percentage of intersects where hyphae were present relative to the total number of intersects counted.

### 2.4 Quantification of *M. hapla* in substrate and plant roots

In order to extract *M. hapla* from the substrate samples (10 g), a density centrifugation method was used, in which the organic particles were floated on Ludox HS-40 (Pfannkuche and Thiel, 1988). The supernatant containing the nematodes was poured through 10 µm sieves, preserved in 4% formaldehyde and stained with a few drops of Rose Bengal and counted under a stereomicroscope (40-fold magnification).

A representative sample was prepared from each washed root system. The roots were cut into 1–2 cm pieces and transferred to a 500 ml polyethylene bottle. The bottles were filled with 250 ml of 1% NaClO solution (Dan Klorix household bleach diluted 1:1.8 with tap water) and shaken at 450 rpm for 3 min. The suspension was passed through a 250 µm sieve to remove the roots and placed over a 10 µm sieve to collect the nematode eggs. The egg suspension on the 10 µm sieve was rinsed with tap water for a few seconds to remove residual NaClO. Eggs were counted under a microscope (100-fold magnification). The number of eggs/g root dry weight was chosen as most accurate parameter for root-knot nematode reproduction and fecundity, as suggested by Abd-Elgawad (1991).

### 2.5 RNA isolation and RNAseq

RNA for RNAseq was extracted using RNeasy Plant Mini Kit (Qiagen, Venlo, The Netherlands) following the manufacturer’s instructions. Leaf RNA was extracted and sequenced for each treatment from three replicates 48 h and 6 d after inoculation.

#### 2.5.1 Library preparation and RNAseq

RNA integrity numbers (RIN) were determined with the Agilent 2100 Bioanalyzer using Agilent RNA Nano 6000 Chips to confirm concentration and integrity of isolated RNA samples. Whole transcriptome sequencing was performed as basis for *de novo* assembly. Therefore, strand-specific, rRNA depleted libraries were constructed based on 500 ng total RNA using the “TruSeq Stranded Total RNA Library Prep Kit with Ribo-Zero Plant” following the manufacturer’s instructions (Illumina, Berlin, Germany) except that enrichment PCR was performed with 11 cycles.

For analysis of differentially expressed genes libraries were constructed based on 800 ng total RNA using the “TruSeq RNA Sample Preparation v2 Kit” following the manufacturers’ instructions (Illumina, Berlin, Germany) except that enrichment PCR was performed with 11 cycles. After equimolar pooling of all libraries (depleted and polyA-enriched), paired end sequencing of 2x100 base pairs was performed in one run on the NextSeq2000 Sequencing System (P2 Flowcell, 400 Mio Reads).

#### 2.5.2 Read data processing, mapping, and gene expression analysis

Raw read data was processed (demultiplexing, adapter masking and main quality score determination) and converted using bcl2fastq conversion tool (Illumina, Berlin, Germany). *De novo* transcriptome assembly was performed using Trinity v2.11.0 with Trimmomatic plugin (Haas et al., 2013). Trimmed reads from three replicates were quantified using Kallisto (Bray et al., 2016) and functional annotations were identified using BLAST (Altschul et al., 1990) to *A. thaliana* reference transcriptome (TAIR, file: TAIR10_pep_20110103_representative_gene_model_updated). The obtained transcripts per kilobase million (TPM) values were used for gene expression analysis in R 4.3.3 (R Core Team, 2023). Principal component analysis (PCA) was calculated over all transcript abundance values from all samples for proof of data similarity. Principal components were generated using ‘prcomp’ (Mardia et al., 1981; Becker et al., 1996; Venables and Ripley, 2002) and main components were plotted using ‘ggplot2’ (Wickham, 2009). Mean TPM values were used to calculate total log2-fold changes (Log2FC) for all treatments compared to the control for both harvesting time points separately. R packages “edgeR” (Robinson et al., 2010) and “limma” (Ritchie et al., 2015) were used to conduct statistical analysis based on raw counts. “exactTest” was used to calculate differences in mean expression of treatments and control and using “topTags” attained p-values that were corrected based on Bonferroni-Hochberg correction for multiple testing. Obtained false discovery rates (FDR) were used to identify differentially expressed genes (FDR < 0.05). To identify relevant plant stress markers and plant defense response, pathway analysis was done with Kyoto Encyclopedia of Genes and Genomes (KEGG, Kanehisa and Goto, 2000). To evaluate the mentioned hypotheses the following assumptions for log2FC changes were made (**Table 1**).

**Table 1:** Parameter setting for changes in log2FC-level.

Hypotheses	Parameters for log2FC
(H1) general stress markers are less responsive in *P. tanacetifolia* in MH+PC than in MH	MH < -1 and MH+PC > MH
	MH > 1 and MH+PC < MH
(H2) *P. chlamydosporia* enhances the plant defense response by additive effects in MH+PC compared to MH and PC	MH+PC < -1 and MH+PC < PC and MH+PC < MH
	MH+PC > 1 and MH+PC > PC and MH+PC > MH

The following test parameters were set to test the stated hypotheses regarding transcriptomic changes in *P. tanacetifolia* caused by *M. hapla* (MH), *P. chlamydosporia* (PC) or the combination of both (MH+PC).

### 2.6 Analysis of plant metabolites

Leaf metabolites were extracted, derivatized and quantified via GC-MS using tissue harvested and shock-frozen after 48 h, 6 d and 28 d of inoculation. Pulverized plant material (5 mg) from six replicates was freeze-dried and processed according to Wegener et al. (2023). Extraction buffer included 10 μM ribitol as an internal standard. Metabolites were separated on a RTx^®^-5MS column (30 m, iD 0.25, df 0.25 μm) using a GC-MS-system consisting of a Trace 1310 GC coupled to a TSQ9000 mass spectrometer (Plassmeier et al., 2007). Sample aliquots (1 μL) were injected in splitless mode into the GC-MS column. The initial temperature was kept at 80°C for 3 min, then raised to 325°C at a rate of 5°C min^-1^ and finally kept at 325°C for 2 min. The MS transfer line and electron impact ion source temperatures were adjusted to 250°C and 220°C, respectively. The spectra were recorded within a scanning range of 50 - 650 mz^-1^ (mass/charge). All substances were compared with reference substances in terms of retention time and mass spectrum. These reference substances are re-measured each time the column is changed in order to adjust the retention times. Quantification was conducted on peak areas of characteristic compound mass/charge ratio normalized to ribitol (at 217 mz^-1^) and to the dry weight of the material. Significant changes in metabolite concentrations were calculated in R 4.3.3 (R Core Team, 2023) based on Kruskal–Wallis test with Dunn’s Test for multiple comparisons with Benjamini and Hochberg (1995) correction as *post-hoc* test.

## 3 Results

### 3.1 Determination of *M. hapla* in substrate and roots

**Figure 1:**
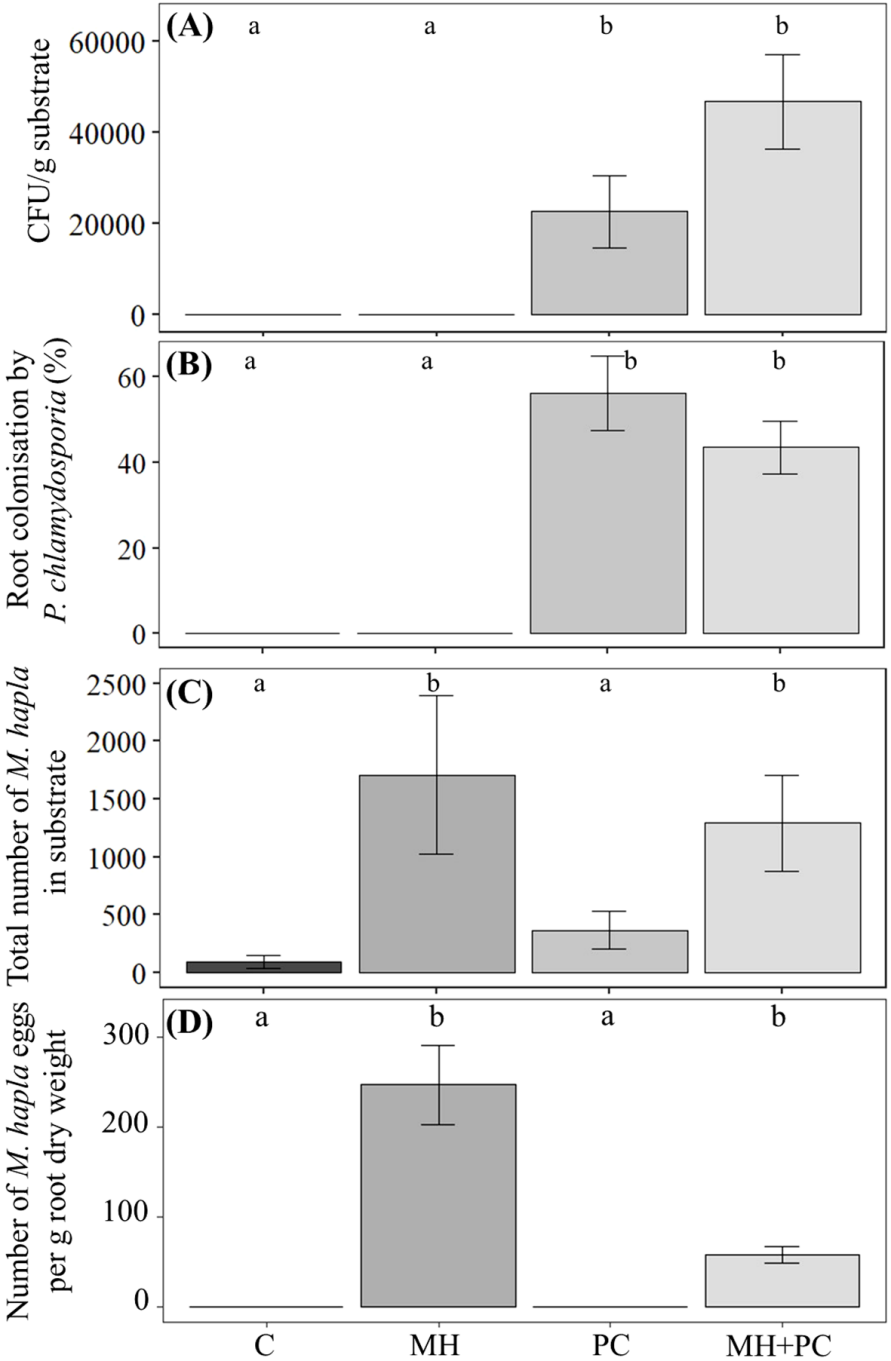
Quantification of *Pochonia chlamydosporia* and *Meloidogyne hapla* in substrate, extent of *P. chlamydosporia* root colonization and amount of *M. hapla* eggs in *Phacelia tanacetifolia* 28 d after inoculation with *M. hapla* (MH), *P. chlamydosporia* (PC) and their combination (MH+PC). **(A)** Colony forming units of *P. chlamydosporia* per g substrate 28 d after inoculation (mean ± SE, n=12). **(B)** Percentage of intersects where hyphae were present relative to the total number of intersects counted for each treatment 28 d after inoculation (mean ± SD, n=12). **(C)** Total number of *M. hapla* in substrate for each treatment 28 d after inoculation (mean ± SE, n=12). **(D)** Number of *M. hapla* eggs per g root dry weight for each treatment 28 d after inoculation (mean ± SE, n=12). C, control; MH, inoculated with *M. hapla*; PC, inoculated with *P. chlamydosporia*; MH+PC, inoculated with *M. hapla* and *P. chlamydosporia*. Different letters indicate significance of difference (Kruskal–Wallis test with Dunn’s *post hoc* test, p < 0.05).

*M. hapla* was quantified 48 h (**Supplementary Figure S1A**), 6 d (**Supplementary Figure S1B**) and 28 d after inoculation. Unexpectedly, nematodes could be found in all substrates 28 d after inoculation (**Figure 1C**). For example, in substrate of treatments C and PC, 95 ± 53 and 369 ± 163 nematodes in total were found, respectively. Compared to treatments C and PC, 18-fold more nematodes were counted in treatments MH (1709 ± 687 nematodes/pot) and 14-fold (compared to the control) in MH+PC (1293 ± 412 nematodes/pot). The amount of *M. hapla* in the treatment MH+PC was 24.3% lower compared to the treatment MH. Additionally, *M. hapla* eggs were extracted from *P. tanacetifolia* roots and quantified 28 d after inoculation (**Figure 1D**). In treatment MH and MH+PC, 247 ± 44 and 58 ± 10 eggs/g root dry weight were found, respectively. As expected, no *M. hapla* eggs were found in C and PC, indicating no nematode reproduction during the experiment. As nematodes were found in the substrate of these treatments, it can be assumed that these were contaminations during the harvest or beginning carry-over from drained irrigation water. Furthermore, replicates with *M. hapla* contamination in C and PC were excluded from the RNAseq approach.

### 3.2 Colony-forming units in the substrate and colonization of plant roots by *P. chlamydosporia*

To evaluate establishment of *P. chlamydosporia* in the substrate, we counted colony-forming units (CFU) at 48 h (**Supplementary Figure S1C**), 6 d (**Supplementary Figure S1D**) and 28 d after inoculation. As expected, *P. chlamydosporia* was absent in treatment C and MH after 28 d. On the other hand, in treatment PC 3.26×10^4^ ± 7.97×10^3^ CFU/g substrate and in MH+PC 4.68×10^4^ ± 1.05×10^4^ CFU/g substrate were counted with no significant difference (**Figure 1A**).

The percentage of intersects where hyphae were present relative to the total number of intersects was determined 28 d after inoculation. Complementary to the CFU results, no hyphae were found in *P. tanacetifolia* roots in treatments C and MH, while roots were colonized by *P. chlamydosporia* in treatments PC and MH+PC by 56.3 ± 8.7% and 43.6 ± 6.2%, respectively, with no significant difference of colonization (**Figure 1B**).

### 3.3 Shoot and root dry weight and nutrient state of *P. tanacetifolia*

After 28 d, the inoculation with MH and/or PC had no effect on *P. tanacetifolia* above ground dry weight (**Supplementary Figure S2A**). In contrast, inoculation of *M. hapla* alone significantly reduced root dry weight by 42% (**Supplementary Figure S2B**). Additionally, there existed no differences for nitrogen, phosphate, and carbon content in *P. tanacetifolia* tissue (**Supplementary Figure S3**).

### 3.4 *P. tanacetifolia* transcriptome changes induced by nematode and fungal inoculation

Trinity assembled a total of 105,493 transcripts and 69,473 genes derived from these transcripts. Functional annotations based on the *Arabidopsis thaliana* reference transcriptome were identified for 21,227 of these genes.

#### 3.4.1 Principal component analysis and differential gene expression

**Figure 2:**
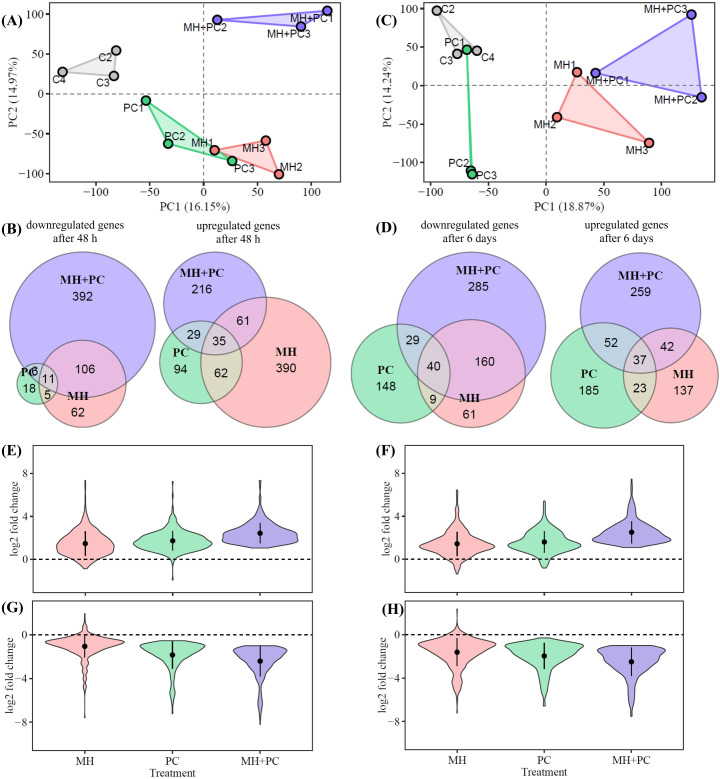
Transcriptome analysis of *Phacelia tanacetifolia* for the systemic effects after inoculation with *Meloidogyne hapla* (MH), *Pochonia chlamydosporia* (PC) and a combination of both (MH+PC). Total leaf RNA was subjected to RNAseq. Principal component analysis (PCA) **(A)** 48 h or **(C)** 6 d after inoculation displays the clustering of the three replicates for each treatment. Specificity and overlap of differentially expressed genes (DEGs) in *P. tanacetifolia* **(B)** 48 *h* and **(D)** 6 d after inoculation are shown in Venn diagrams (FDR < 0.05, upregulated: log2FC > 1 or downregulated log2FC < -1, n = 3). To globally investigate the additive effect of the combined inoculation (hypothesis 2), upregulated transcripts of MH+PC (FDR < 0.05, log2FC > 1) were compared to single inoculated treatments (Kruskal–Wallis test with Dunn’s *post hoc* test, p < 0.001), **(E)** 48 h (341) or **(F)** 6 d (390) after inoculation and downregulated transcripts of MH+PC (FDR < 0.05, log2FC < -1) were compared to single inoculated treatments **(G)** 48 h (515) or **(H)** 6 d (514) after inoculation.

For an overview, TPM values were subjected to principal component analysis (PCA). The PCA for both time points showed a reliable clustering of the replicates, with an obvious treatment-dependent pattern. This clear difference between the control (C) and all other treatments reflects the impact of *M. hapla* and *P. chlamydosporia* on the transcriptome of *P. tanacetifolia*. After 48 h, MH and PC conditions were not entirely separated from each other, but they clearly differed from C and especially from the combination MH+PC (**Figure 2A**). A similar treatment-specific clustering is visible 6 d after inoculation. An overlap appears between C and PC due to high variance for PC, while treatments MH and MH+PC lack an overlap and clearly separated from C and PC (**Figure 2C**).

For the 48 h and 6 d time points, 1539 and 1573 transcripts showed differential accumulation, respectively (**Figures 2B, D**). The largest portion of differentially expressed genes (DEGs) occurred with MH+PC, namely 392 decreased and 216 increased transcripts after 48 h, and 285 decreased and 259 increased transcripts after 6 d. However, MH had a stronger effect on the shoot transcriptome at the early time point of 48 h than after 6 d, since 548 transcripts revealed increased abundance after 48 h and 239 transcripts after 6 d. In contrast, PC-treatment effects become more apparent with increasing treatment duration (**Figures 2B, D**). A transcript comparison of the MH+PC exposure to the individual exposure regimes revealed a stronger overlap between MH and MH+PC, especially for transcripts with reduced expression for both treatments. But the most surprising observation was that the combined treatment MH+PC altered a very large set of unique transcripts, and this was already apparent at the early time point of 48 h when the *P. chlamydosporia* growth was still very low.

Furthermore, the comparison between MH+PC and the single inoculated treatments underlines the additive effect of the combined inoculation. Transcripts that were regulated in MH+PC were less altered in MH and PC. Overall, this effect applied to 37.9% downregulated and 30.3% upregulated transcripts for both harvest times (**Figures 2E–H**; **Supplementary Table S1** for downregulated transcripts and **Supplementary Table S2** for upregulated transcripts).

**Figure 3:**
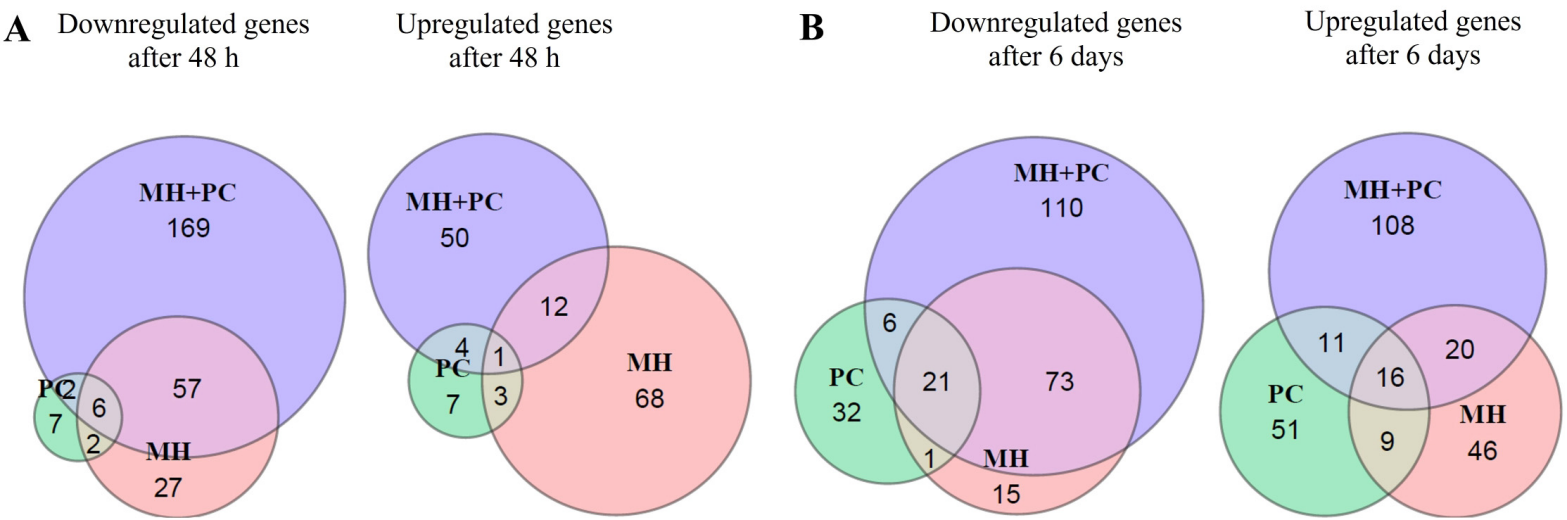
Analysis of functionally annotated transcripts of *Phacelia tanacetifolia* for the systemic effects after inoculation with *Meloidogyne hapla* (MH), *Pochonia chlamydosporia* (PC) and a combination of both (MH+PC). Leaf RNA was subjected to RNAseq. Specificity and overlap of differentially expressed genes (DEGs) in *P. tanacetifolia* **(A)** 48 *h* and **(B)** 6 d after inoculation are shown in Venn diagrams (FDR < 0.05, upregulated: log2FC > 1 or downregulated log2FC < -1, n = 3).

About 40% of the *P. tanacetifolia* DEGs could be functionally annotated with *Arabidopsis thaliana* as reference (**Figure 3**). Repeating the analysis with the functionally annotated transcripts did not change the proportions of DEGs and overlap of DEGs between the treatments to any major extent. Therefore, the following in-depth analysis of individual transcripts was carried out with functionally annotated transcripts to assign functional dependencies.

**Figure 4:**
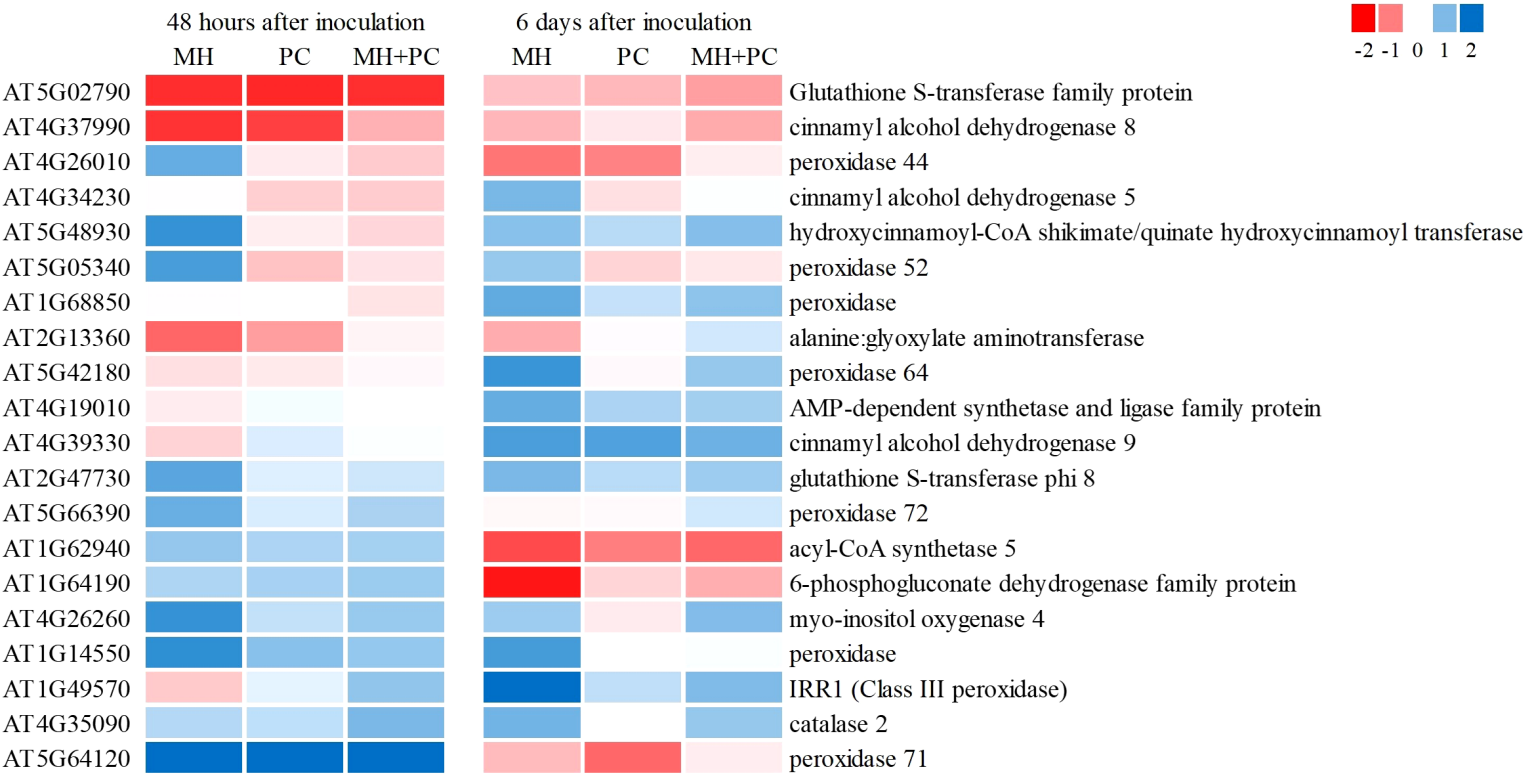
Heatmap of stress markers in *Phacelia tanacetifolia* in response to *Meloidogyne hapla* (MH), *Pochonia chlamydosporia* (PC) and a combination of both (MH+PC). Heat maps with log2-fold changes of transcripts that were less regulated in MH+PC compared to MH (hypothesis 1: MH > 1 and MH+PC < MH or MH < -1 and MH+PC > MH) for one or both harvesting time points (**Supplementary Table S3**). KEGG pathways: phenylpropanoid biosynthesis (ath00940), glutathione metabolism (ath00480), ascorbate and aldarate metabolism (ath00053), and peroxisomes (ath04146).

In total, 20 transcripts related to general stress markers fitted the parameters for H1 and were less regulated in MH+PC compared to MH (**Figure 4**; **Supplementary Table S3**). After 48 h, *CINNAMYL ALCOHOL DEHYDROGENASE 8*, *ALANINE: GLYOXYLATE AMINOTRANSFERASE* and *GUTATHIONE-S-TRANSFERASE L3* (*GSTL3*) were less downregulated in MH+PC compared to MH, while among other peroxidases (*PER44*, *52*, *71* and *72*) and *MYO-INOSITOL OXYGENASE 4* (*MIOX4*) were less upregulated. After 6 d, *ACYL-CoA SYNTHETASE 5*, *PER44* and *6-PHOSPHOGLUCONATE DEHYDROGENASE* were less downregulated in MH+PC compared to MH, while *CINNAMYL ALCOHOL DEHYDROGENASE 5* and *9*, *PER64* and *CATALASE* 2 (*CAT2*) were less upregulated. Contrary to H1, 29 transcripts were stronger regulated in MH+PC compared to MH. In more detail, 9 transcripts decreased more in MH+PC compared to MH, including for example *GSTU7*, *8*, *25*, and *GSTF2* and *ASCORBATE PEROXIDASE 1* and *2* (*APX1* and *2*), while 20 transcripts were stronger upregulated, including for example *PER 2*, *16*, *56*, *GSTF3* and *9* and *MIOX1* (**Supplementary Table S4**).

**Figure 5:**
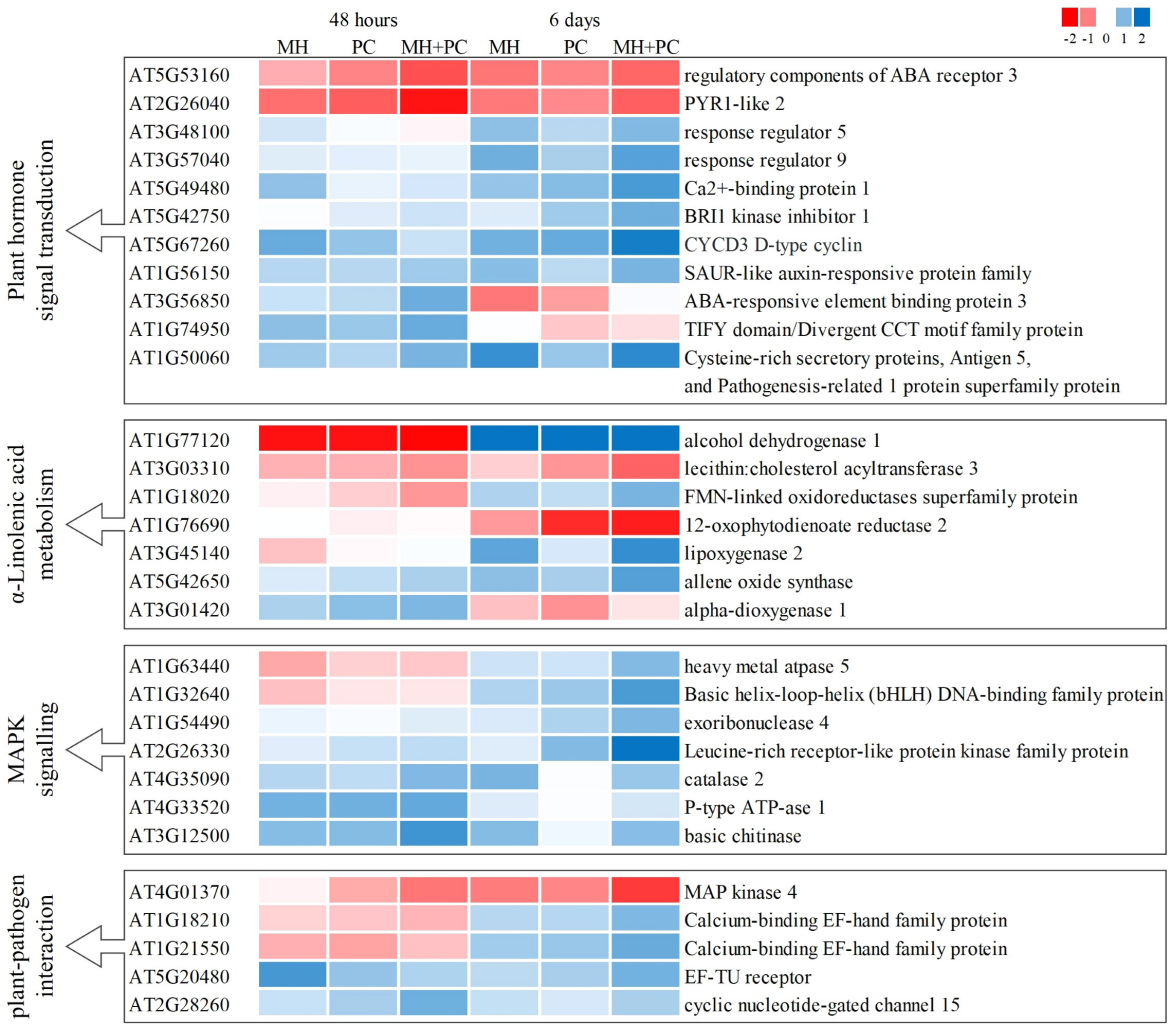
Heatmap of transcripts related to plant defense signaling in *Phacelia tanacetifolia* in response to *Meloidogyne hapla* (MH), *Pochonia chlamydosporia* (PC) and a combination of both (MH+PC). Heat maps with DEGs (FDR < 0.05) for plants inoculated with *M. hapla* (MH) or *P. chlamydosporia* (PC) or with a combination of both organisms (MH+PC) in comparison to uninoculated control plants. Heatmaps show log2-fold changes for treatments compared to the control for harvest 48 h and 6 d after inoculation fitting the parameters of hypothesis 2. KEGG pathways: Plant hormone signal transduction (ath04075), α-Linolenic acid metabolism (ath00592), MAPK signaling pathway (ath04016), Plant-pathogen interaction (ath04626).

As assumed in H2, the combined inoculation MH+PC could enhance the plant defense response due to synergistic regulation of defense-related transcripts. Several transcripts with a role in abscisic acid (ABA) and jasmonic acid (JA) signaling, as well as transcripts involved in plant-pathogen interactions and MAPK signaling responded stronger in the combined treatment than in the single inoculation. Examples for this type of response were *PYRABACTIN RESISTANCE 1-LIKE 2* (*PYL2*), *12-OXOPHYTODIENOIC ACID REDUCTASE 2* (*OPR2*), *LIPOXYGENASE 2* (*LOX2*)*, CAT2* and *MITOGEN ACTIVATED PROTEIN KINASE 4* (*MPK4*) (**Figure 5**; **Supplementary Table S5**).

Additionally, as stated above, it appeared interesting that the total number of DEGs was in all cases higher in MH+PC compared to MH and/or PC, except of upregulated transcripts 48 h after inoculation, where MH treatment had a higher impact on plant transcriptomic changes (**Figure 3**). DEGs mainly regulated in MH treatment were for example disease resistance proteins, *LACCASEs*, and *ACC OXIDASE 5* but also membrane proteins, cell wall localized proteins, peroxidases and proteins involved in inositol metabolism (**Supplementary Table S6**) and *WALL-ASSOCIATED KINASE 2* and *5* (*WAK2* und *5*) were downregulated in PC and MH+PC (6 d) but not in MH. A detailed list of downregulated and upregulated transcripts mainly for MH, PC or MH+PC is available in the supplements (**Supplementary Table S6** for MH, **Supplementary Table S7** for PC and **Supplementary Table S8** for MH+PC).

### 3.5 Treatment effects on plant metabolites

**Figure 6:**
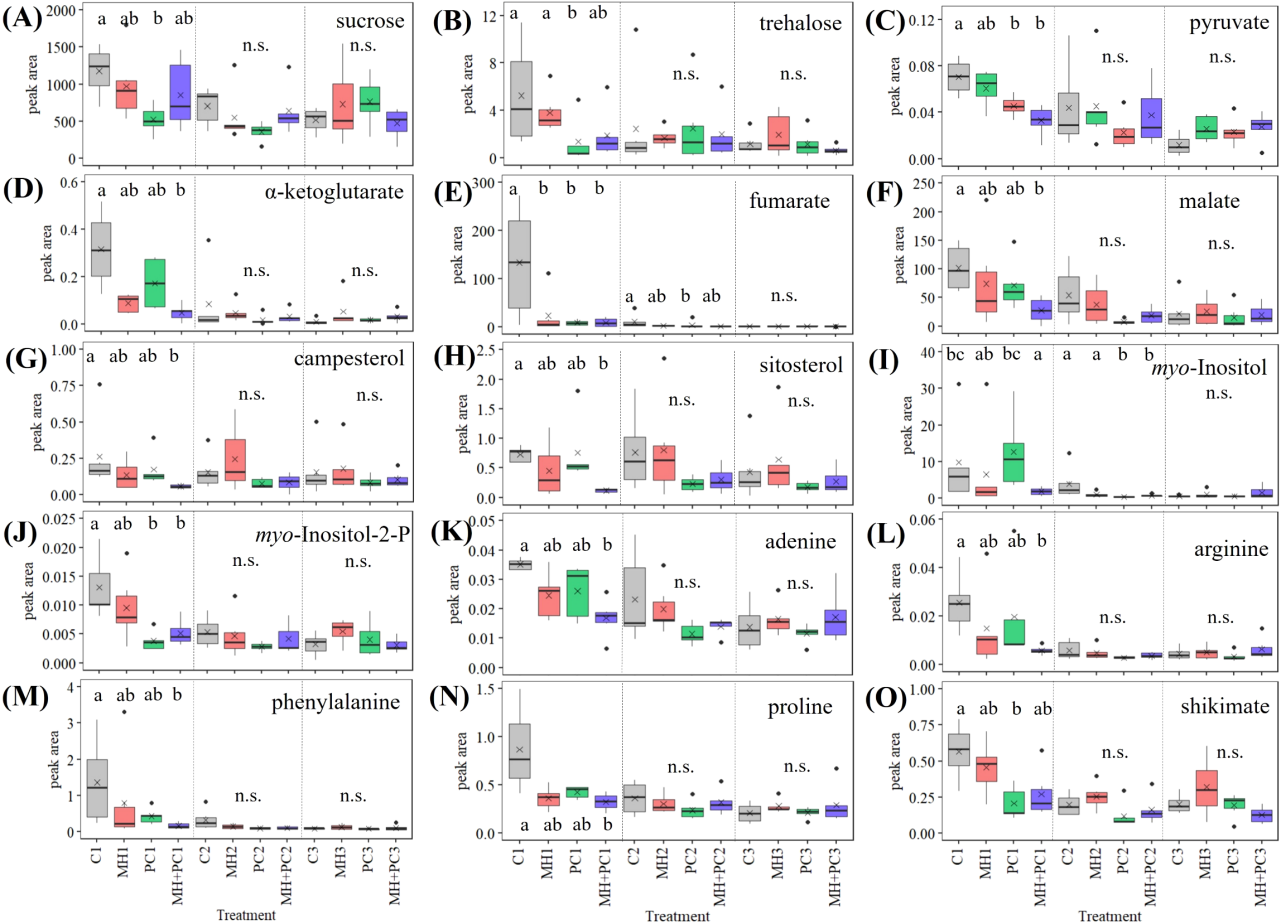
Relative amounts of selected *Phacelia tanacetifolia* metabolites from central sugar metabolism **(A–C)**, citric acid cycle **(D–F)**, phytosterols **(G, H)** and myo-inositol **(I, J)**, amino acids **(K–N)** and shikimate **(O)** in response to *Meloidogyne hapla* (MH), *Pochonia chlamydosporia* (PC) and a combination of both (MH+PC). Box plots show the peak areas of characteristic compound mass/charge ratio of the metabolites normalized to ribitol (217 mz-1) and leaf dry weight. The treatments with *M. hapla* (MH), with *P. chlamydosporia* (PC) or with a combination of both organisms (MH+PC) were compared with the uninoculated control plants (48 h after inoculation = C1, MH1, PC1, MH+PC1; 6 d after inoculation = C2, MH2, PC2, MH+PC2; 28 d after inoculation = C3, MH3, PC3, MH+PC3). The horizontal line in the boxes indicates the median and x the mean (n=6). Values with no letter in common are significantly different (Kruskal–Wallis test with Dunn’s Test for multiple comparisons with Benjamini and Hochberg (1995) correction as *post-hoc* test, p < 0.05). n.s. indicates no significant differences between the treatments for the respective harvesting time point.

In order to assess changes in metabolism as ultimate process in response to the treatments, a metabolome analysis complemented the transcriptome assessment. The GC-MS-based analysis covered metabolites from central and specialized metabolism. The most conspicuous observation concerned the fact that significant differences between the treatments almost only occurred 48 h after inoculation (**Figure 6**). The metabolite contents at the later time points of 6 d and 28 d were usually lower than in the younger developmental stage.

PC treatment lowered the contents of the disaccharides sucrose (α, β-1,2-Glc-Fru) and trehalose (α-1,1-Glc-Glc) compared to control plants. The trend of decreased amounts of sucrose and trehalose in the treated plants after 48 h was mimicked by several other metabolites of the central energy pathways, namely pyruvate, α-ketoglutarate, and malate. And also, the amino acids arginine, phenylalanine and proline, and shikimate, the intermediate for synthesis of aromatic amino acids and diverse specialized metabolites, followed this pattern.

The analysis of phytosterols showed significant differences between the treatments exclusively after 48 h in *P. tanacetifolia* leaf tissue (**Figures 6F, G**). Treatment MH+PC led to significantly lower campesterol and sitosterol content in *P. tanacetifolia* leaves compared to control plants after 48 h.

The in part severe and differentiated effect of treatments on metabolite contents at the early time point of inoculation was not seen at the later time points of 6 d and 28 d, with the exception of *myo*-inositol and fumarate (**Figure 6**). Overall, the pronounced treatment-induced perturbation of metabolism after 48 h was followed by a homeostatic state of leaf metabolism irrespective of the treatment at later time points.

## 4 Discussion

This study on the commonly used cover crop *P. tanacetifolia* explores the systemic response of the shoot to infection of the roots with *M. hapla* or *P. chlamydosporia*, and the combination of both. The ultimate goal of this research and possible application in the field is to establish a *P. chlamydosporia* population in the cover crop to reduce the number of the root-knot nematode *M. hapla* in the soil. The hypothesis is that following ploughing under of the cover crop, the presence of *P. chlamydosporia* will lower the nematode pressure during cultivation of the main crop (Uthoff et al., 2023). Apart from testing this system in field trials, there is the need to understand the interference between *M. hapla*, *P. chlamydosporia* and *P. tanacetifolia.* Therefore, we set up this study to sensitively monitor changes in *P. tanacetifolia* following the single or combined inoculation. We sought answers to two questions, namely whether *P. chlamydosporia* inoculation poses a stress to the plant and whether and how the combined treatment affects metabolism and gene expression? Plant responses to stress combinations have moved into the focus of current research on abiotic and biotic plant-environment interactions in order to bridge lab experiments and field experiments (Dietz and Vogelsang, 2024; Kumar et al., 2024). To this end we chose the systemic response as appropriate readout.

Growth data revealed that after 28 d, the shoot biomass was indistinguishable between all treatments including the control. This kind of no-effect was observed before for example upon inoculating six parsley genotypes irrespective whether classified as good or no host plants (Noskov et al., 2024). However more commonly, strong prevalence of root nematodes in the field drastically reduces crop yield, for example in soybeans (Saha et al., 2023). In a converse manner to the no-effect on shoots, the root biomass dropped to 41% in the *M. hapla*-infested plants. Presumably, the soil was a rich substrate, and the reduced root growth had no effect on the shoot. This explanation could be tested by decreased fertilization. The *P. chlamydosporia*-treatment insignificantly lowered the root biomass to 91% of the control. *P. chlamydosporia* inoculation often has no effect or even a beneficial effect on biomass production of plants including cover crops (Coutinho et al., 2021).

Simultaneous addition of *P. chlamydosporia* reduced the *M. hapla*-induced root growth inhibition to 67% after 28 d. In order to avoid this late stage of infection and progressed damage, we opted for an early time point of 48 h and a later time point of 6 d for the analysis of transcripts and metabolites where still the nematodes are in the second juvenile stage (J2) (Dávila-Negrón and Dickson, 2013). Nevertheless, after 28 d the tendency of the beneficial effect of *P. chlamydosporia* on root health was shown in the combined treatment. Furthermore, in treatments inoculated with *P. chlamydosporia* and *M. hapla* the CFU/g substrate were higher while root colonization was lower compared to treatments inoculated with *P. chlamydosporia* alone. This indicates that *P. chlamydosporia* as nematophagous fungus prefers to feed on nematodes instead of growing endophytically in plant roots and, therefore, supports plants by reducing the number of nematodes in the soil. Uthoff et al. (2023) already confirmed the potential of *P. chlamydosporia* isolate P001 as a biocontrol agent against *M. hapla* if inoculated to *P. tanacetifolia*. In addition, they found that plants inoculated simultaneously with *P. chlamydosporia* and *M. hapla* had a higher root dry weight 50 d after inoculation compared to plants only inoculated with *M. hapla*. In this study, the amount of *M. hapla* and the root dry weight was measured 28 d after inoculation. This indicates that significant effects on the *M. hapla* population and the resulting effects on root weight might only be found after a longer period of time. Furthermore, it is likely that studies on multiple interacting organisms will not result in strongly significant differences as the establishment and growth of the individual organisms depends on several factors that additionally interact with each other, resulting in a highly complex system.

### 4.1 Less response of ROS-related general stress markers in leaf tissue of the combined treatment

As starting point for this study, we hypothesized that *P. tanacetifolia* exposed to the combination of the nematode *M. hapla* and the fungus *P. chlamydosporia* encounters less stress even in the early stages of infestation due to the antagonistic interaction between *P. chlamydosporia* and *M. hapla*. We focused on transcripts coding for markers of general abiotic and biotic stress related to phenylpropanoid biosynthesis, glutathione metabolism, ascorbate and aldarate metabolism, and peroxisomes. Indeed, these transcripts accumulated less in the shoot of plants exposed to the combined treatment compared to the single treatment with *M. hapla* (H1). Nematode infestation triggers ROS- and redox-related responses in the roots of the host plants (Denjalli et al., 2024). Both stimulatory and inhibitory effects at the site of infestation have been described, in particular recent research identified the inhibitor Mj-NEROS that interacts with the RIESKE center of the photosynthetic electron transport chain and suppresses ROS generation in the plastids of roots and leaves (Stojilković et al., 2024). Published research mostly concerned the local response of the infestation site. Here we focused on the systemic effect of root infestation on the shoot.

Both microorganisms, *M. hapla* and *P. chlamydosporia*, induce ROS production while interacting with the plant root (Baker and Orlandi, 1995; Lamb and Dixon, 1997; Mata-Pérez and Spoel, 2019). Heme peroxidases (PER) exist as a huge gene family and the responsive PER transcripts showed no clear pattern regarding the treatments and harvesting time points. As the nature of the electron donor for PER depends on the structure of the enzyme (Apel and Hirt, 2004) and class III PER are known to be involved in abiotic and biotic stress responses (Kidwai et al., 2020), it can be assumed that during infection a variety of systemic ROS-related reactions take place.

Within the redox regulatory network, the cytosolic *APX1* and *APX2* were stronger downregulated in treatments with a combined inoculation of *P. chlamydosporia* and *M. hapla* compared to control at both harvesting time points. APXs efficiently detoxifies H_2_O_2_ using ascorbate as electron donor and is a central component of the ROS gene network in different subcellular compartment. Its expression is often enhanced in response to abiotic and biotic stress such as pathogen attack (Davletova et al., 2005). The lower amounts of cytosolic APX in the combined treatment indicates, contrary to H1, an antagonistic effects of *P. chlamydosporia* on *M. hapla*-induced plant damage and suppression of plant defense response with systemic effects on the shoot.

Another example is *MIOX4* that was stronger upregulated in *M. hapla* compared to both treatments inoculated with *P. chlamydosporia*. MIOX4 is the first enzyme in the inositol route to ascorbate and important for syncytium development of sugar beet nematode *Heterodera schachtii* (Siddique et al., 2009). An *A. thaliana* quadruple *myo*-inositol oxygenase mutant showed a significant reduction in susceptibility to *H. schachtii* and the primary function of *myo*-inositol oxygenase for syncytium development is assumed to be the reduction of *myo*-inositol levels and thereby a decrease in the galactinol level to avoid the induction of defense-related genes (Siddique et al., 2014). This is in line with this study as *M. hapla* and the combined treatment with *P. chlamydosporia* and *M. hapla* decreased *myo*-inositol content, while *P. chlamydosporia* alone did not affect the *myo*-inositol content. Liu and Chen (2003) described that excluding *myo*-inositol from the medium increased *P. chlamydosporia* conidia formation which could be a reason *MIOX4* is less upregulated in plants with potentially endophytic fungal growth. Here it remains unclear, if the slight upregulation of *MIOX4* is actively caused by the fungus or if it is a plant response to support fungal endophytic growth.

### 4.2 Synergistic effects of *P. chlamydosporia* and *M. hapla* intensify plant defense

Previous studies categorize *P. chlamydosporia* as endophyte with neutral or positive effects on plants like tomato, *Arabidopsis*, or barley (Escudero and Lopez-Llorca, 2012; Larriba et al., 2015; Schouten, 2016; de Souza Gouveia et al., 2019). In line with these findings, the second hypothesis addressed the presumed stimulatory effect of *P. chlamydosporia* on activating defense responses, helping the plant to better defend against the nematode in the combined treatment with *M. hapla* and *P. chlamydosporia*.

Defense responses of plants are often regulated on long distance by phytohormones, namely salicylic acid (SA), jasmonic acid (JA), ethylene (ET) and abscisic acid (ABA). For example, LOX2 that is part of the JA-pathway and α-Dioxygenase 1 (DOX1) that is induced in response to SA, and different transcripts related to the ABA pathway were stronger regulated in the combined treatment compared to single inoculated treatments (**Supplementary Table S5**). ABA plays an important role in the preinfectional defense of *A. thaliana* against *M. paranaensis* as their population was reduced by over 50% with the exogenous application of ABA 24 h before the nematode inoculation even though ABA had no toxic effect on the nematodes *in vitro* (Yop et al., 2023).

Besides ABA, JA is well described to be involved in pathogen defense (Halim et al., 2006) and the resistance against root-knot nematode of the genus *Meloidogyne* (Fan et al., 2015; Medeirosa et al., 2015; Bali et al., 2018; Gouveia et al., 2023). Furthermore, JA signaling is crucial for the establishment of mutualistic interactions between plants and microorganisms, including bacteria and fungi (Hause and Schaarschmidt, 2009), also shown for *P. chlamydosporia* (Zavala-Gonzalez et al., 2017). Our study underlines the systemic importance of phytohormone signaling, as several transcripts related to their metabolism were systemically regulated in leaves.

### 4.3 The phenomenon of early metabolic disturbance

Most metabolic effects were exclusively detected 48 h after inoculation and also in the combined treatment with *M. hapla* and *P chlamydosporia* (**Figure 6**). Only the combined inoculation with *P. chlamydosporia* and *M. hapla* led to significantly lower adenine, arginine, phenylalanine, and proline as well as campesterol and sitosterol contents compared to control plants. It is important to note here again, that we focused on systemic effects caused either by long distance signaling from the infested root to the shoot or by increased sink strength (Zhou et al., 2023).

Pyruvate is metabolized to ethanol and serves as precursor of acetyl-CoA, the acetyl moiety of which enters the citric acid cycle. The combined treatment significantly decreased the content of α-ketoglutarate and malate compared to the control plants after 48 h, while fumarate was significantly decreased in all treatments after 48 h, and also in plants treated with *P. chlamydosporia* 6 days after inoculation. Fumarate and malate have a variety of functions in biochemical pathways in plants and fumarate acts as an alternative carbon sink for photosynthates similar to starch (Araújo et al., 2011). The reduction of fumarate and malate content in *P. tanacetifolia* leaves by *P. chlamydosporia* and *M. hapla* could be caused due to consumption of carbohydrates by both inoculated organisms which establish additional sinks in the plant root.

Additionally, several amino acids function as C- and N-source and are metabolized by *P. chlamydosporia*. However, isolates differ in their effectiveness of carbon source utilization (Vieira dos Santos et al., 2019). The here studied *P. chlamydosporia* isolate P001 remains unexplored regarding its utilization of carbon sources. This metabolome analysis may give a first hint, but detailed investigations are needed. On the one hand, external application of amino acids, especially phenylalanine, can reduce the number of galls caused by root knot nematode *M. javanica* in tomato roots (Amdadul Hoque et al., 2014). On the other hand, low amino acid availability to the nematode reduces susceptibility of roots to *M. incognita* infestation (Dutta et al., 2024). The reduction of amino acids in plant leaf tissue could also be a plant response to suppress *M. hapla* growth, or amino acids could be metabolized by *P. chlamydosporia*, or most probably a combination thereof that lead to a synergistic effect of the combined inoculation. The metabolome data (**Figure 6**) suggest that the bipartite and tripartite interaction challenge plant homeostasis systemically only at an early stage. Later on, the plant returns to a homeostatic metabolism. This conclusion is supported by the observation that above-ground biomass was indifferent between the various treatments.

*M. hapla* and *P. chlamydosporia* invade the root cells of plants and secrete different molecules and proteins to support this process by destabilizing the root cell wall and membrane, and suppressing the plant defense (Abad and Williamson, 2010; Larriba et al., 2014; Shukla et al., 2018; Pentimone et al., 2019). Additionally, *P. chlamydosporia* secrets chitinases, chitin deacetylases and a chitosanase during nematode egg parasitism (Aranda-Martinez et al., 2016), which may also interact with nearby plant roots and target cell wall-related processes. The *WAK2* and *WAK5* were downregulated in the combined treatment and the single inoculation with *P. chlamydosporia*. WAKs are candidates as polygalacturonic acid receptors and are induced by SA. *M. hapla* releases polygalacturonases during infection (Jaubert et al., 2002), but for *P. chlamydosporia* it is not described if it releases polygalacturonases while different plant pathogenic fungi do (Nakamura and Iwai, 2019).

The effector proteins of *P. chlamydosporia* and *M. hapla* trigger local responses and likely initiate systemic plant signaling processes that explain the transcriptional and metabolic responses of the leaves.

## 5 Conclusions

In general, the plant response to plant-parasitic nematodes and endophytic fungi differs strongly. In our experimental setup, *P. chlamydosporia* had neither a negative nor a positive effect on shoot or root growth. In the combined treatment with *M. hapla*, however, a positive effect of *P. chlamydosporia* on root growth was detected. It is noteworthy that in many cases the regulation of transcripts is stronger in the combined treatment compared to the individually inoculated treatments. Therefore, the combined treatment with *M. hapla* and *P. chlamydosporia* had a partially additive effect on the transcriptome and metabolome of the plant as suggested in hypotheses 2. This effect is beneficial if it concerns relevant defense genes. Hypotheses 1 was supported by 20 transcripts related to general stress markers as these transcripts were less abundant in *P. tanacetifolia* in the combined treatment compared to the treatment only with *M. hapla*. Therefore, both hypotheses seem to be at least partly confirmed. But, the exact mechanism of interaction between *P. chlamydosporia*, *M. hapla* and *P. tanacetifolia* remains largely unknown and needs further research. The co-inoculation of *P. chlamydosporia* and *M. hapla* in this study and the resulting variation in expression patterns and regulations underline the complexity of trophic interaction. Especially the here demonstrated systemic response including phytohormones and ROS as long-distance signaling offers interesting research perspectives as the cross-talk between different phytohormones as well as the specific function of peroxidases still leaves many questions unanswered. Even though potentially contaminated replicates were excluded from RNA sequencing, further studies are warranted with strict regime that avoid any nematode contaminations with consequently proper analyses of the RNAseq in control and *P. chlamydosporia* treatments.

In an agricultural context, the understanding of these interactions could improve the application of *P. chlamydosporia* as a biocontrol agent against *M. hapla*. To improve the establishment of the biocontrol agent, several ingredients can be added. For example, amino acids, phytohormones and/or nutrients that support *P. chlamydosporia* performance as well as ingredients that suppress *M. hapla* growth such as chitinases, chitin deacetylases and chitosanases which naturally decompose the nematodes’ cuticula. Nonetheless, abiotic parameters in the field must be taken into account since they affect the conditional expression of the plant (defense) response. In conclusion, this study underlines the systemic response of plants to microorganisms entering the plant roots.

## Data Availability

The raw data supporting the conclusions of this article will be made available by the authors, without undue reservation. RNA-seq data will be available after acceptance at NCBI Sequence Read Archive SRA - NCBI (nih.gov), BioProject ID: PRJNA1205911, available online at http://www.ncbi.nlm.nih.gov/bioproject/1205911.
