# Supplementary material for: Pochonia chlamydosporia synergistically supports systemic plant defense response in Phacelia tanacetifolia against Meloidogyne hapla
Source: Front Plant Sci. 2025 Jan 16;15:1497575. doi: 10.3389/fpls.2024.1497575 (PMC11779738; doi:10.3389/fpls.2024.1497575)
Supplement: Supplementary file 1 [file DataSheet1.docx]

Supplementary Material

# Supplementary Figures


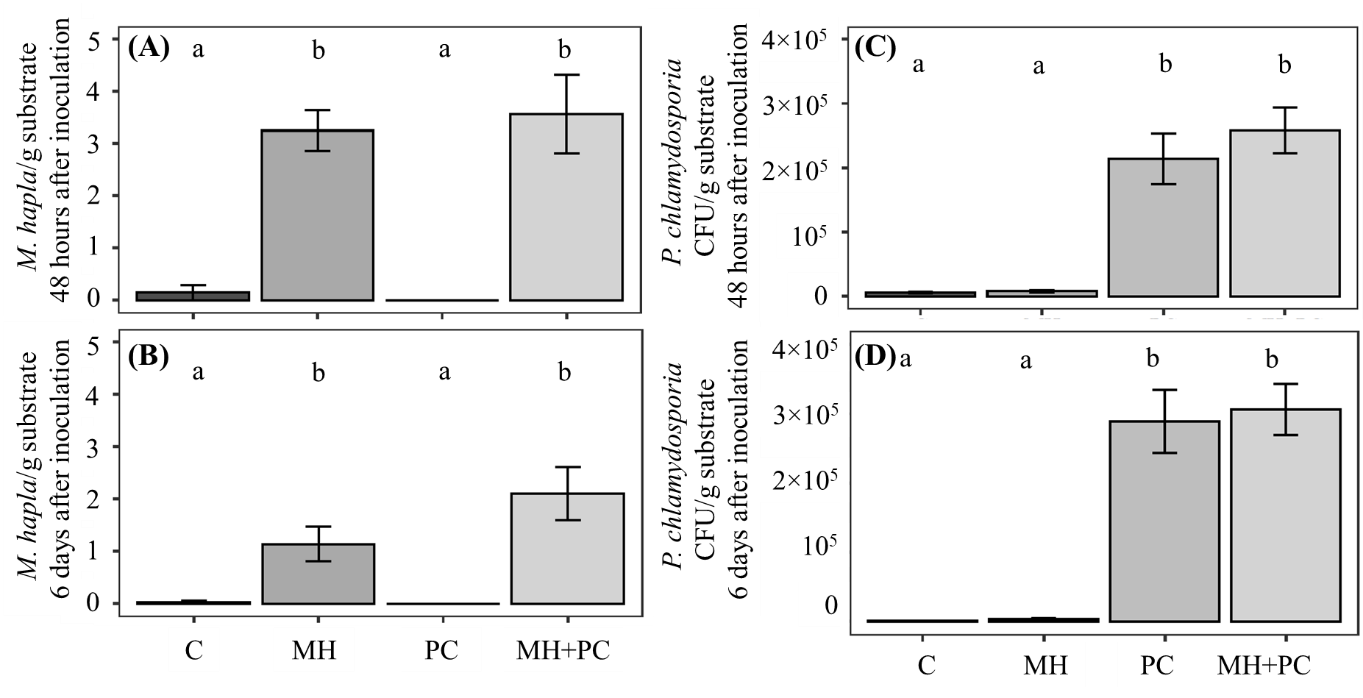


**Figure S1: Quantification of *Pochonia chlamydosporia* and *Meloidogyne hapla* in substrate 48 hours (A, C) and 6 days (B, D) after inoculation with *M. hapla* (MH), *P. chlamydosporia* (PC) and their combination (MH+PC).** **(A)** Total number of *M. hapla* per g substrate for each treatment 48 h after inoculation (mean ± standard error, n=12). **(B)** Total number of *M. hapla* per g substrate for each treatment 6 d after inoculation (mean ± standard error, n=12). **(C)** Colony forming units (CFU) of *P. chlamydosporia* per g substrate for each treatment 48 h after inoculation (mean ± standard error, n=12). **(D)** Colony forming units of *P. chlamydosporia* per g substrate for each treatment 6 d after inoculation (mean ± standard error, n=12). All treatments were conducted in 400 g substrate per pot. Values with no common letter are significantly different (Kruskal–Wallis test with Dunn’s *post hoc* test, p < 0.05).


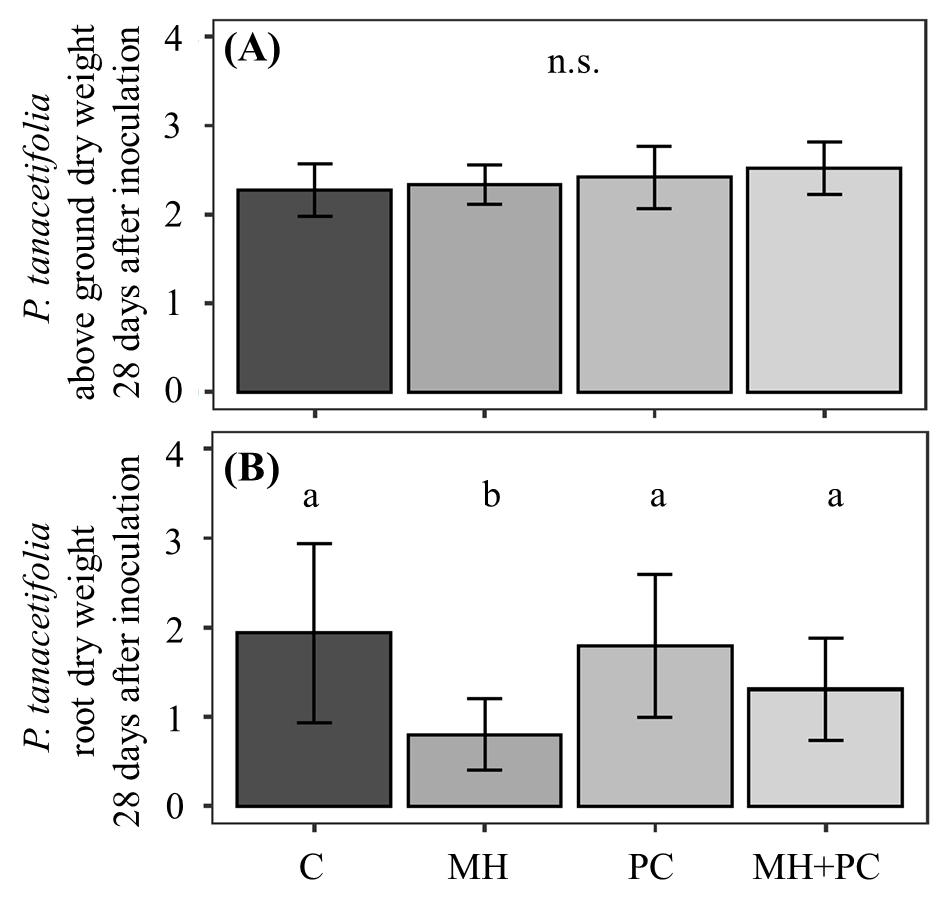


**Figure S2: Above ground and root dry weight of *Phacelia tanacetifolia* 28 days after inoculation with *Meloidogyne hapla* (MH), *Pochonia chlamydosporia* (PC) and their combination (MH+PC).** **(A)** Above ground dry weight of *P. tanacetifolia* 28 d after inoculation (mean ± standard deviation, n=12). **(B)** Root dry weight of *P. tanacetifolia* 28 d after inoculation (mean ± standard deviation, n=12). Values with no common letter are significantly different (Kruskal–Wallis test with Dunn’s *post hoc* test, p < 0.05). n.s. indicates no significant differences between the treatments.


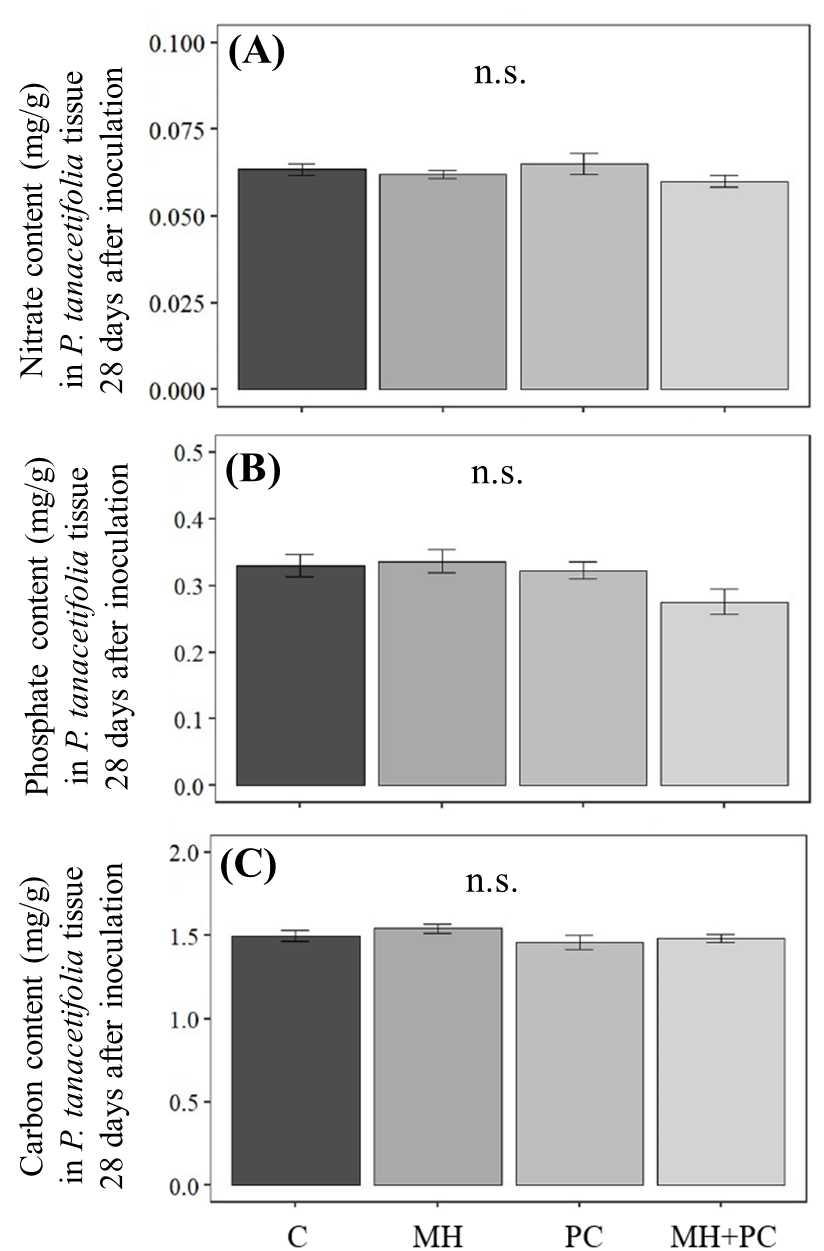


**Figure S3: Nutrient contents in *Phacelia tanacetifolia* tissue 28 days after inoculation with *Meloidogyne hapla* (MH), *Pochonia chlamydosporia* (PC) and their combination (MH+PC).** The above-ground plant material was dried for 5 d at 40 °C, weighed and the above-ground plant material was pulverized with a vibrating mill (Retsch MM 400) at 20 Hz for 2 mins. The nitrogen and carbon content was determined using an elemental analyzer (Unicube, Elementar Analysensystem GmbH, Langenselbold, Germany). The phosphate content was determined using high-temperature oxidation and colourimetric quantification according to Watanabe and Olsen (1965). The phosphate concentration was analyzed using FOW injection analysis (FIA-Lab II, MLE GmbH, Dresden, Germany) at 880 nm. **(A)** Nitrate content in *P. tanacetifolia* 28 d after inoculation (mean ± standard error, n=12). **(B)** Phosphate content in *P. tanacetifolia* 28 d after inoculation (mean ± standard error, n=12). **(C)** Carbon content in *P. tanacetifolia* 28 d after inoculation (mean ± standard error, n=12) Comparison between all treatments showed no significant differences (n.s. indicates no significant differences between the treatments, Kruskal–Wallis test, p > 0.05).
